# A Bionic Flapping Magnetic‐Dipole Resonator for ELF Cross‐Medium Communication

**DOI:** 10.1002/advs.202403746

**Published:** 2024-06-14

**Authors:** Zhi Cheng, Jing Zhou, Bin Wang, Qiong Wu, Liang Ma, Zhi Qin, Jie Shen, Wen Chen, Wei Peng, Jianglei Chang, Penghong Ci, Shuxiang Dong

**Affiliations:** ^1^ School of Materials Science and Engineering Wuhan University of Technology Wuhan 430070 China; ^2^ Institute for Advanced Study Shenzhen University Shenzhen 518061 China; ^3^ Electronic Materials Research Laboratory, Key Laboratory of the Ministry of Education, School of Electronic Science and Engineering Xi'an Jiaotong University Xi'an 710049 China; ^4^ State Key Laboratory of Superlattices and Microstructures Institute of Semiconductors Chinese Academy of Sciences Beijing 100083 China

**Keywords:** cross‐medium magnetic communication, electric‐mechano‐magnetic coupling, ELF antennas, high efficiency resonators

## Abstract

Extremely low‐frequency (ELF) electromagnetic (EM) waves adeptly propagate in harsh cross‐medium environments, overcoming rapid decay that hinders high‐frequency counterparts. Traditional antennas, however, encounter challenges concerning size, efficiency, and power. Here, drawing inspiration from nature, we present a groundbreaking piezo‐actuated, bionic flapping‐wing magnetic‐dipole resonator (BFW‐MDR), operating in the electro‐mechano‐magnetic coupling mechanism, designed for efficient ELF EM wave transmission. The unique rigid‐flexible hybrid flapping‐wing structure magnifies rotation angles of anti‐phase magnetic dipoles by tenfold, leading to constructive superposition of emitted magnetic fields. Consequently, the BFW‐MDR exhibits a remarkable quality factor of 288 and an enhanced magnetic field emission of 514 fT at 100 meters with only 6.9 mW power consumption, surpassing traditional coil antennas by three orders of magnitude. The communication rate is doubled by the ASK+PSK modulation method. Its robust performance in cross‐medium communication, even amidst various interferences, underscores its potential as a highly efficient antenna for underwater and underground applications.

## Graphical abstract

This study introduces a piezo‐actuated, bionic flapping‐wing magnetic‐dipole resonator (BFW‐MDR) for efficient ELF electromagnetic wave transmission, utilizing electro‐mechano‐magnetic coupling. The innovative design significantly enhances magnetic field superposition and rotation angle, boosting emission efficiency. With exceptional quality factor and low power consumption, the BFW‐MDR excels in air‐seawater communication, promising potential for underwater and underground applications.

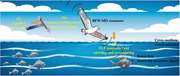

## 1 Introduction

Communication within subaquatic and subterranean environments, as well as air‐to‐underwater and underground cross‐medium communication, encompasses vital applications in positioning, search and rescue, mining, and unmanned vehicle control. Conventional cross‐medium communication techniques,^[^ ^1^, ^2^, ^3^, ^4^, ^5^ ^]^ such as hydroacoustic, underwater optical, and high‐frequency electromagnetic wave communication, encounter challenges in severe channel conditions, including substantial attenuation in diverse lossy medium and notable skin effect losses, restricted cross‐medium communication range. In contrast, extremely low‐frequency (ELF) electromagnetic (EM) waves (ranging from 3 to 30 Hz) with wavelengths spanning from 100 000 to 1000 000 km demonstrate minimal attenuation, robust penetration capabilities through various mediums, and exceptional resistance to interference, thereby facilitating efficient cross‐medium information transmission.^[^ ^6^ ^]^

The transmission of ELF EM waves using traditional coil antennas encounters constraints associated with their considerable size, diminished radiation efficiency, and elevated power consumption. To overcome these impediments, proposals have been set forth to explore mechanically oscillating magnetic dipole moments as potential solutions, incorporating electromagnetic‐motor‐driven rotating magnets and acoustic/piezoelectric resonators.^[^ ^7^, ^8^, ^9^, ^10^, ^11^, ^12^, ^13^, ^14^, ^15^, ^16^, ^17^, ^18^, ^19^ ^]^ While the former can generate ELF time‐varying magnetic fields (B‐field) with robust radiation capability, electromagnetic motors contend with substantial mechanical size, elevated power consumption, and the generation of electromagnetic interference, thus limiting efficient information modulation.^[^ ^20^, ^21^, ^22^ ^]^ In contrast, magnetoelectric (ME) coupled linear resonators operating in the longitudinal mode, a subtype of conventional piezoelectric resonators, offer advantages such as reduced size, swifter response, and enhanced resistance to electromagnetic interference.^[^ ^23^, ^24^ ^]^ However, the inherent challenge faced by piezoelectric actuators in efficiently oscillating the magnetic dipoles of magnetostrictive materials significantly hampers the radiation capability of the B‐field.^[^ ^25^, ^26^, ^27^ ^]^ Addressing this challenge, the latest electro‐mechanical‐magnetic (EMM) coupled swinging resonator,^[^ ^28^ ^]^ specifically a piezo‐actuated cantilever beam featuring a step‐stiffness junction and a tip magnet mass operating in bending mode, has embraced mechanical swinging of magnetic dipole moments, resulting in highly efficient and stronger B‐field radiation. Nevertheless, the presence of piezoelectric ceramics and the beam substrate imparts substantial inherent stiffness to the step‐stiffness cantilever beam, thereby constraining further optimal amplification of the oscillation angle of the magnetic dipoles.

In this study, we propose an innovative solution: Bionic Flapping‐Wing Magnetic Dipole Resonator (BFW‐MDR). The bionic flapping‐wings in this resonator are one pair of elastic beams equipped with permanent magnets. The two flapping‐wings are symmetrically connected at actuating end of the piezoelectric composite beam by flexible, but robust films (acting as flexible hinge); and synchronously, they are reinforced by one pair of magnetic repulsion forces facing outside by another pair of magnets with the same magnetization direction attached at the piezo‐beam. This magnetic repulsive force reinforced‐flexible hinge design exhibits with the rigid‐flexible (step‐stiffness) junction character, but it is more rotational compliance, effectively mitigating the bending stiffness of the elastic beam wings and providing substantial freedom for the flapping oscillation of magnetic dipoles under piezoelectric actuating. The synchronized flapping vibration of the two wings with anti‐phase magnetic dipoles oscillation further facilitates the constructive superposition of the B‐field emitted from each wing, significantly amplifying the transmission of ELF EM waves. As a result, the proposed BFW‐MDR enhances the Electro‐Mechano‐Magnetic (EMM) coupling capability, demonstrating a high‐quality factor (∼288), ultralow power consumption (only 6.9 mW), and a three‐order‐of‐magnitude increase in efficiency for emitting ELF EM waves compared to conventional loop coil antennas. Furthermore, an ASK + PSK modulation method was designed to substantially reduce the relaxation time, resulting in an improved communication rate of 2 bps. Additionally, cross‐medium communication tests were conducted between air and seawater under various external interferences, showcasing the robustness of the BFW‐MDRs for underwater and underground communications in harsh environments.

## 2 Results

### 2.1 Design and Mechanism Analysis of Bionic Flapping‐Wing Magnetic‐Dipole Resonator (BFW‐MDR)

**Figure 1:**
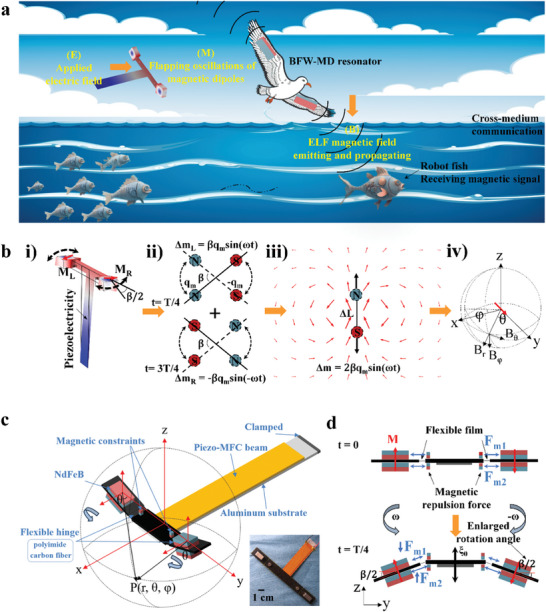
Principle diagram of the BFW‐MDR for cross‐medium magnetic field communication. a) Application scenarios illustrating Extremely Low‐Frequency (ELF) cross‐medium communication, and B‐field emitting mechanism of the electro‐mechano‐magnetic (EMM) coupled resonator model. b) Panels (i)–(iii) depict the vibrational modes of the resonator, the magnetic dipole superposition effect of two wings, and B‐field emitting in three dimensions space, respectively. c) Schematic diagram of the bionic flapping‐wing resonator, depicting the cantilever piezo‐beam and the two wings connected by flexible hinges. The permanent magnets on each wing exhibit opposite magnetization directions. Small permanent magnets, sharing the same magnetization direction as the magnets at the end of each wing, are strategically positioned at the beam‐wing joint, serving as “magnetic constraint” for the wings. The inset is the device's physical photograph. d) Detailed schematic of the flexible hinges and magnetic constraints, showcasing the amplification of the swing angle of the magnetic dipole and the achievement of constructive superposition of B‐field.

Classical Maxwell's equations elucidate the duality between magnetic field and electrical field, allowing mechanical antennas to emit electromagnetic waves through the motion, rotation, or oscillation of electric or magnetic dipole moments. In nature, avian flight patterns exemplify that a flapping wing mode induces the rotation of both wings from a swinging motion to an out‐of‐plane direction, minimizing energy consumption upon achieving a steady flight state.^[^ ^29^, ^30^ ^]^ Inspired by avian flapping motion, we propose a bionic flapping‐wing magnetic‐dipole resonator (BFW‐MDR) designed to exhibit low power consumption and high magnetic field radiation via electro‐mechano‐magnetic (EMM) coupling. This resonator can efficiently achieve Extremely Low‐Frequency (ELF) cross‐medium communication with interference immunity and high penetrability, as depicted in **Figure** **1**a. When applying a programmable alternating electric field (**E**) to piezoelectric composite beam, as shown in Figure 1b‐i, the vibration of the cantilever beam induces the flapping oscillation of the two symmetrically distributed permanent magnets (M_L_ and M_R_) positioned at the end of the wings. In Figure 1b‐ii, the electromechanical coupling effect of piezoelectric and permanent magnets further induces the oscillation of inside magnetic dipole moments (**m**). This oscillation gives rise to the Extremely Low Frequency (ELF) magnetic field (**B**) emitting at the intrinsic frequency of the structure. The magnetic dipoles of the two wings demonstrate a superposition process of magnetic field vectors attributable to the opposite phase of oscillations due to the contrary direction of magnetization, as illustrated in Figure 1b‐ii. Figure 1b‐iii,iv illustrates the radiation process of the magnetic field into space by the flapping magnetic dipole.

According to the magnetic dipole oscillation model, the B‐field in the near field (kr ≪ 1) is given by:^[^ ^28^ ^]^

(1) $$B=\frac{\mu_{o}\Delta m}{4\pi r^{3}}\;\left(2\cos\theta \hat{r}+\sin\theta \hat{\theta }\right)$$

The magnetic fields of the oscillating magnetic dipoles at the near‐field distance (r) is

(2) $$\left|B_{r}\right|=\left|\frac{\mu_{o}\Delta m\;}{2\pi r^{3}}\cos\theta \right|$$

The magnetic dipole moment transformation (Δ*m*) is defined as Δ*m*  = *B_r_* *V_m_sin*β/μ_0_, where B_r_ is the remanent magnetization of the permanent magnets, V_m_ is the volume of the permanent magnets, and β is the oscillation angle of the permanent magnets.

Equation (2) discloses that increasing the oscillation angle β augments the magnetic field strength in the radial direction (r). Traditionally, radiation enhancement has been achieved by constructing piezoelectric composite beams with stiffness gradients to amplify the end oscillation angle or by grouping individual array elements through intricate phase matching. However, the incorporation of piezoelectric ceramics and the beam substrate contributes to an overall increase in beam stiffness. Consequently, the design of stiffness gradients is unable to effectively alleviate the substantial inherent beam stiffness, thereby significantly limiting the oscillation angle of the magnetic dipole.^[^ ^28^, ^31^ ^]^ Regarding the approach of grouping individual arrays, the strength of the emitted B‐field can be enhanced when superimposing two magnetic moment vectors with opposite magnetization directions and oscillation phases, based on the superposition principle of magnetic field.^[^ ^32^, ^33^ ^]^ Otherwise, the superimposition of two magnetic moment vectors with mismatched phases may reduce the total emitting strength of the B‐field.

To surmount these challenges and achieve radiation enhancement, we drew inspiration from the wing flapping behavior of birds, leading to the design of the BFW‐MDR to amplify the oscillation angle and ensure phase matching. The device, illustrated in Figure 1c, comprises a piezoelectric composited cantilever beam (main body) with components such as a Macro Fiber Composite (MFC), an aluminum substrate, carbon fiber wings, and NdFeB permanent magnets. The MFC, a flexible piezoelectric actuator composed of piezoelectric ceramic fibers and polymers, is affixed to the aluminum substrate to form the piezoelectric composited cantilever beam (termed as piezo‐beam). One side of the piezo‐beam serves as the clamping end, while the other side serves as actuating end, where the two connected carbon fiber wings through one pair of flexible hinges (made of polyimide film) are actuated for generating symmetrically flapping oscillation. To reinforce hinge support and prevent gravity‐induced sagging of the two large magnets on wings, one pair of small permanent magnets, sharing the same magnetization direction as large magnets at the end of each wing, are strategically positioned at the joint of the piezo‐beam and the wings for producing magnetic repulsion forces (MRF). This MRF reinforced flexible film hinge exhibits with rotational compliance character due to effectively mitigated bending stiffness at the rigid‐flexible (step‐stiffness) joint, providing substantial freedom for the flapping oscillation of magnetic dipoles through piezoelectric actuating.

Figure 1d shows a flapping oscillation period (T) of the two wings. In the initial status (t = 0 T), the flexible hinge‐MRF constraint establishes a mechanical equilibrium, keeping the two wings in horizontal plane, therefore, the rotational‐angle β/2 = 0 for the wings and the piezo‐beam. When the piezo‐beam experiences a displacement (ξ_0_) in the z‐axis via piezoelectric actuating under an applied voltage, the up magnetic repulsion force F_m1_ and down magnetic repulsion force F_m2_ become unbalanced. At t = T/4, the piezo‐beam goes up with a displacement ξ_0_, resulting in F_m1_ < F_m2_. This imbalance further leads to an accelerated flapping movement of the wings. At t = T/2, the flapping displacement reaches its maximum due to the lagging phenomenon of the two wings behind the piezo‐beam. Because of minimizing the inertia force of the two wings at t = T/2, consequently, rotational angle of β/2 also synchronously reaches its maximum value. Hence, the incorporation of flexible hinge and MRF constraints is more efficient method for generating larger flapping oscillations of the magnetic dipoles via piezo‐beam actuating, significantly intensifying the Electro‐Mechano‐Magnetic (EMM) coupling effect. Furthermore, to ensure the superposition of vector magnetic fields generated by the magnetic dipoles on each wing, the permanent magnets on the two wings are designed to have opposite magnetization directions and oscillation phases. This biomimetic approach aligns the oscillating phases of the magnetic dipoles on the two wings, leading to a constructive superposition of the B‐field radiation.

In this resonant system, the vibration of the piezoelectric beam induces oscillation in the attached permanent magnet, where the former drives the motion of the latter. Both elements are integral to the resonator's functionality. However, it is important to distinguish between the mechanical quality factor (Q_m_) of the piezoelectric resonator and the quality factor (Q) of the magnetic dipole antenna.

The evaluation of resonant coupling in a resonant system is closely associated with the high mechanical quality factor (Q_m_) of the composite material, typically determined by the 3 dB bandwidth of the resonant peaks. When the flapping‐wing resonator functions as a magnetic dipole mechanical antenna, we utilize the static magnetic field theory proposed for near‐field conditions to evaluate the communication efficiency in magnetic near‐field communication. A system characterized by a high‐quality factor (high‐Q) indicates minimal mechanical energy loss and an enhanced resonant state response. As for an EMM coupling system, a high‐Q means it is more conducive to achieving strong flapping oscillations, therefore, a high‐efficiency B‐field radiation. The quality factor (Q) is defined as^[^ ^34^, ^35^ ^]^

(3) $$Q=\omega \frac{max\left\{W_{m},W_{e}\right\}}{P_{\mathrm{Loss}}}$$

where *W_m_* is the magnetic field energy stored in space, *W_e_* is the electric field energy stored in piezoelectric dielectric medium, and *ω* and *P_Loss_* are circular frequency, and the internal power losses (ohmic loss and mechanical loss) of the resonator, respectively. From the equation^[^ ^28^ ^]^

(4) $$Q=\frac{{\left(\alpha B_{r}V_{m}\right)}^{2}}{9V_{a}\mu_{0}Ct_{p}^{2}\mathrm{PF}}$$

In Equation (4), the parameter (α) is defined as the piezo‐actuation coefficient (β  =  α*E*), signifying that an increase in the resonator's driving electric field *E* can enhance the permanent magnet rotation angle *β*. *C* is the capacitance of the piezoelectric phase, *t_p_* is the interdigital electrode spacing in piezoelectric MFC actuator, *V_a_* is the volume of antenna, and (PF) is the power factor. The detailed derivation is available in Section S1 (Supporting Information).

A high piezo‐actuation coefficient (α), strong magnetization (M), larger permanent magnet volume (V_m_), and reducing PF (power factor, indicative of lower losses and a higher mechanical quality factor, Q_m_) collectively contribute to an augmented quality factor Q. Subsequently, adhering to the principle of designing high radiation efficiency through the enhancement of α and β, we undertake a theoretical and experimental validation to analyze the effects of the piezoelectric beam resonant modes on wing vibrations and electromagnetic wave radiations.

### 2.2 BFW‐MDR Vibration Modes Analysis for Rigid Connections/Magnetic Constraints

**Figure 2:**
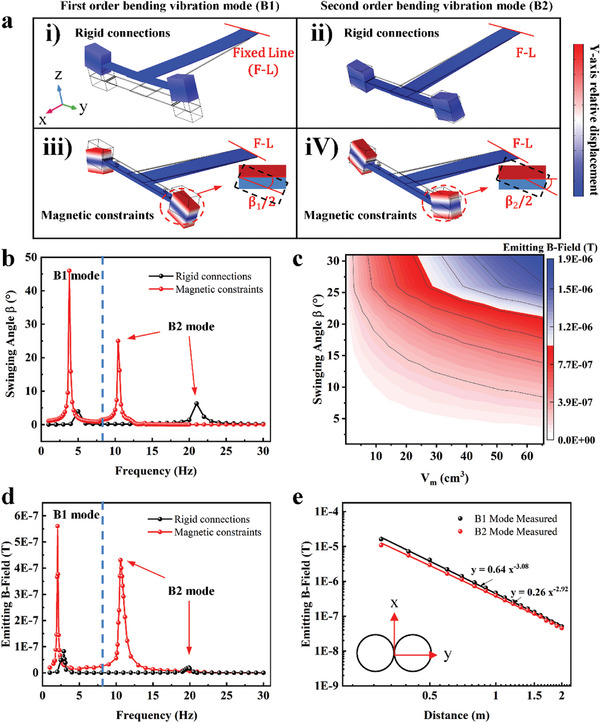
Vibration modes of the BFW‐MDR and analysis of wings‐piezobeam rigid connections and flexible hinge‐magnetic constraints. a) The first and second‐order bending vibration modes of a rigid and magnetically constrained wing‐piezobeam connections. The insets show the oscillation angle β of the two vibration modes. b) The frequency‐domain plots of rotating angle β for the two types of connections simulated by the FEM. c) The calculated magnetic field strength at a distance of 1 m as a function of the volume V_m_ and permanent magnet rotation angle. d illustrates the frequency domain performance of magnetic field emission for both rigid connections and magnetic constraints structures. e) shows the distance decay of B‐field emitting for the device.

As mentioned previously, the design incorporating a flexible hinge and magnetic constraints can amplify the oscillation angle of the magnetic dipoles, resulting in enhanced B‐field radiation. To investigate this phenomenon, finite element analysis (FEM) was employed to compare the flexible hinge‐magnetic force constraint connection and conventional rigid connection methods, and their impact on the swinging angle of the two wings, as depicted in **Figure** **2**a. The FEM model comprises a PZT‐5H piezoelectric ceramic (with mechanical properties similar to piezoelectric MFC), an aluminum alloy beam, and a NdFeB permanent magnet. The joints connecting the wings and the cantilever piezo‐beam are designated as rigid connections (Figure 2a‐i,ii) and magnetic constraints (Figure 2a‐iii,iv). The first‐order bending mode represents the overall up‐and‐down oscillation of the piezo‐beam relative to its fixed point along the Z‐axis, which further induces flapping oscillations in both wings. Intriguingly, in the second‐order bending mode, the permanent magnets exhibit an inward rotation in the yz‐plane. The inset in Figure 2a illustrates the oscillation angle of the two wings for the two vibration modes, revealing a notably larger oscillation angle using the magnetic constraints compared to the rigid connections in both resonant modes. Further calculations of the BFW‐MDR's oscillation angles in the frequency domain for the two types of wing‐beam connections are depicted in Figure 2b. The implementation of a magnetic constraints manifests a tenfold angle amplification effect, serving as a crucial assurance for the BFW‐MDR to achieve a magnetic field emission intensity.

Typically, electromagnetic waves below 3 Hz hold potential for applications in neurotherapy within the medical domain. However, signal transmission can be influenced by diverse natural activities. Consequently, a second‐order resonance mode with the resonant frequency exceeding 3 Hz is employed in designing the BFW‐MDR. This design objective is geared toward seamlessly amalgamating heightened electromagnetic radiation capabilities with elevated communication rates.

To substantiate the assertion that an increased rotation angle of the magnetic dipole enhances the propagating magnetic field strength, Figure 2c presents simulation results of B‐field emitting, the relationship between the magnetic volume V_m_, swinging angle β of the permanent magnet, and the spatial magnetic flux density. In the resonant mode, the permanent magnet undergoes repetitive rotation around x‐axis. By manipulating the rotation angle β and volume V_m_ of the permanent magnet, we measured the magnetic field strength at a distance of 1 meter along the Y‐axis. The rotational angle β of the permanent magnet is directly proportional to the magnetic flux density, and an augmentation in the volume V_m_ of the permanent magnet corresponds to an increase in spatial magnetic field strength. Figure S1a (Supporting Information) provides the spatial distribution of the magnetic field for a rotating permanent magnet, while Figure S1b (Supporting Information) depicts the time‐domain relationship between magnetic flux density and rotation angles. The Root Mean Square (RMS) value of the magnetic field strength is $1/2\sqrt{2}$ of the peak‐to‐peak value, confirming that the flapping wing's increased rotation angle results in a higher magnetic field strength.

In accordance with Equation (2), the maximum magnetic field strength produced by the rotating magnetic dipole is situated in the radial r‐direction, corresponding to the y‐direction. Therefore, given the opposite oscillating phases, implementing the reverse magnetization scheme for the two wings becomes pivotal to maximize the synergistic effect. Figure 2d presents the magnetic field strength plotted against frequency for a reverse‐magnetized rigidly connected and magnetically constrained flapping‐wing resonator. The swept‐frequency magnetic field strength is measured along the y‐direction at a 1 m distance from the resonator. We compared the magnetic field radiation performance of the two structures under the same driving electric field. The experimental results closely align with the simulation data, indicating that the rigid attachment of the magnet restricts the magnetic field emission performance. Conversely, the magnetically confined structure significantly enhances the magnetic field's emission, achieving a several‐fold amplification effect. Due to the reverse magnetization, the first‐order bending mode displays a larger rotation angle, leading to a higher magnetic field strength at the first‐order resonance frequency of 2.2 Hz. This observation aligns with the greater swing angle of the first‐order bending vibration determined through finite element calculation. Additionally, angular displacements of the permanent magnet operating in the first‐ and second‐order resonance modes were recorded using a laser displacement sensor, as depicted in Figure S2a (Supporting Information). In the first‐order bending resonance, the permanent magnet on the flapping wings produces a rotational angle displacement of 40° (±20°) around x‐axis, while the second‐order bending resonant mode can produce a 20° angular displacement. Subsequently, the generated magnetic field strength of the tapped resonator as a function of emitting distance was compared for the two resonant modes, as illustrated in Figure 2e. The inset exhibits the radiation direction of the reverse‐magnetized flapping wing resonator, highlighting the synergistic enhancement of magnetic field strength in the y‐direction due to the opposite magnetization direction and rotation phase of the two wings. Furthermore, as indicated in Figure S2b (Supporting Information), a circular search coil was positioned at a 1 m distance from the pair of flapping wing resonators to gauge the radiated magnetic field strength of the antenna. According to Faraday law of electromagnetic induction, the received voltage signal is *V_r_ = N·dΦ/dt = 2πf·N·S·B*, where *f* is flapping wing's resonant frequency, *N* and *S* are turn number and receive area of search coil, respectively. *V_r_* exhibits a sinusoidal pattern akin to the excitation signal, suggesting that the ELF B‐field generated by the bionic flapping wing magnetic‐dipole resonator (BFW‐MDR) possess the capability to propagate over extended distances. We also constructed the opposite magnetization direction flapping resonator as a comparison, and rigorously validated the exceptional performance of the bionic flapping resonator introduced in this study, and the detailed experimental results are provided in the Section S4 (Supporting Information).

In a subsequent investigation, a flapping wing structure with second‐order bending modes was employed, incorporating reverse magnetization. This strategy ensures the synergistic enhancement effect of the two wings alongside the practicality of the communication frequency.

### 2.3 Near‐Field Radiation Characteristics of the Flapping Magnetic Dipole Resonator

In pursuit of enhancing the radiation efficiency and ability of the extended flapping wing structure, an augmented angle of rotation for the biplane permanent magnet can be achieved by applying a tip mass at the actuating end of the piezo‐beam. Simultaneously, this addition facilitates a stepless regulation of the resonant frequency of the device. Figure S4a (Supporting Information) depicts the time‐domain plot of the received signal *V_r_* from the receiving coil after the incorporation of the tip mass. By adjusting the position of the tip mass in the cantilever piezo‐beam leads to an augmentation in the radiated magnetic field strength of the flapping‐wing resonator. This increase is attributed to the tip mass amplifying the rotation angle of the two wings, as shown in Figure S4b (Supporting Information). Simultaneously, efficient resonance can be achieved within the intrinsic frequency range of 6.86 to 10.7 Hz.

**Figure 3:**
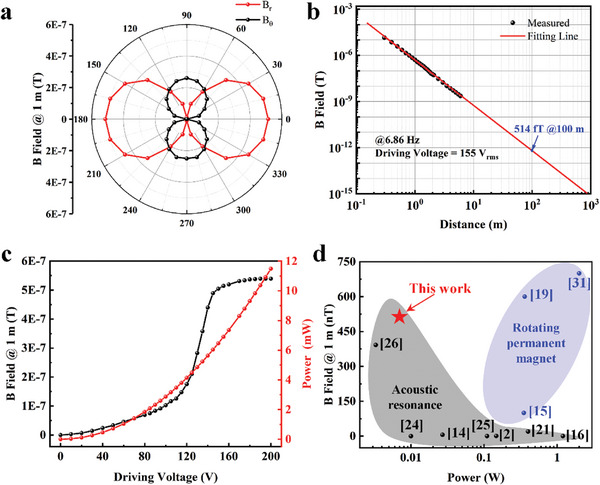
Near‐field radiation characteristics of the BFW‐MDR. a) The radiation pattern of the BFW‐MDR on the yz plane, b) measured and predicted the near‐field radiation intensity of the BFW‐MDR as a function of the emitting distance r, while the far‐field magnetic field strength is predicted through nonlinear fitting, c) the relationship between the voltage and the radiation magnetic field strength/power consumption, d) comparative analysis of the magnetic field strength and power consumption of a representative set of mechanical resonators/antennas.

To assess the directional nature of the B‐field radiation from the mechanical antenna and ensure optimal reception of a stronger magnetic field, we measured the B_r_ value (radial component) and B_θ_ (azimuthal component) at a distance of 1 m from the mechanical antenna with search coil. As depicted in **Figure** **3**a, the mechanical antenna displays an eight‐shaped radiation pattern in the yz plane. Additionally, the maximum intensity of the radiation field is observed in the direction where the receiving antenna and the mechanical antenna are at 0 degrees, i.e., along y‐axis direction.

Figure 3b illustrates the decay curve of the radiated magnetic field strength versus distance for the flapping‐wing magnetic‐dipole resonator. The measurement shows that the emitted B‐field is as high as 514 nT in the y‐axis direction at a distance of 1 m from the resonator (as a mechanical antenna) under a drive voltage of 155 V(rms) at the resonant frequency of 6.86 Hz that corresponds to an input power as low as 6.9 mW. This observation aligns with the radiative decay model for the magnetic dipole, displaying consistent behavior. Based on this data, it is deduced that at the distance of 100 meters, the resonator yields a magnetic field strength of 514 fT. It is expected that cross‐medium magnetic communication with a range of over 100 meters can be achieved. This substantial increase in the operational range of the mechanical antenna presents a promising solution for cross‐medium communication with extremely low power consumption, expanding its range of applications. Figure 3c illustrates the correlation between the radiated field strength and the power consumption of the flapping resonator. As the input voltage increases, the piezoelectric MFC generates a larger strain, causing the two wings flapping with a greater rotation amplitude, nonlinearly increasing the intensity of the radiation field. This nonlinearity arises from the innovative introduction of magnetic confinement and hinge connections. In the resonator's flexible structure, the oscillation angle of the permanent magnet lags behind the voltage changes due to inertia. As the piezoelectric amplitude increases, this lag causes a nonlinear effect between the drive voltage and the radiated magnetic field. The detailed analysis procedure can be viewed in Section S6 (Supporting Information). Concurrently, the power consumption of the resonator rises with increasing voltage. To balance performance with longevity, we maintain the driving electric field of the resonator at ≈0.32 kV mm^−1^. This strategy not only reduces power consumption but also achieves higher radiation efficiency, effectively extending the operating life and stability of the device. Worth noting is that the power consumed by the piezoelectric material is minimal due to the low operating frequency. At a 1‐meter distance, the ratio of magnetic field strength to power is 74.49 nT/mW, representing the highest performance from the piezoelectricity‐based mechanical antennas.

The radiation performance of the BFW‐MDR antenna underwent evaluation by comparison with loop coil antennas of comparable dimensions (19 cm^3^). The detailed analysis of the process can be found in the Section S7 (Supporting Information). When the coil emits a magnetic field at an extremely low frequency of 6.86 Hz, maintaining the same input power (0.0069 W) as the BFW‐MDR, it generates only a 13.8 nT magnetic field strength at a distance of 1 meter. In contrast, achieving the same transmitting magnetic field strength of 514 nT at 1 meter, as accomplished by the BFW‐MDR, requires an input power up to 33 W to operate a conventional loop coil antenna, which is 4782 times higher than that required for BFW‐MDR. These findings highlight that the radiation efficiency of the BFW‐MDR is three orders of magnitude greater than that of a loop coil antenna with equivalent size.

In Figure 3d, we compare the radiated magnetic field strength of the BFW‐MDR with other mechanical antennas: the rotating permanent magnet mechanical antenna and the acoustic resonance mechanical antenna. Our mechanical antenna, utilizing piezo‐actuated flapping wings with magnetic force reinforced bionic flexible hinge to amplify the rotational angle of the permanent magnets, attains a radiated field strength of 514 nT at a distance of 1 meter, the highest radiation strength from the existing acoustic resonance based mechanical antennas. As per Equation (5), the flapping‐wing mechanical antenna demonstrates an impressive quality factor of 288 and exhibits a low power consumption of only 6.9 mW.

### 2.4 Demonstration of Anti‐Interference and Stability of Cross‐Medium Communication

To demonstrate the operational stability of the flapping wing resonator and its potential for practical applications, we conducted a series of signal immunity experiments alongside communication cycle stability tests. These experiments were aimed at assessing the robustness of the ELF magnetic signals emitted by the BFW‐MDR. We explored practical application scenarios to validate the potential applications of ELF cross‐medium communication by detecting signals from the receiving coils.

**Figure 4:**
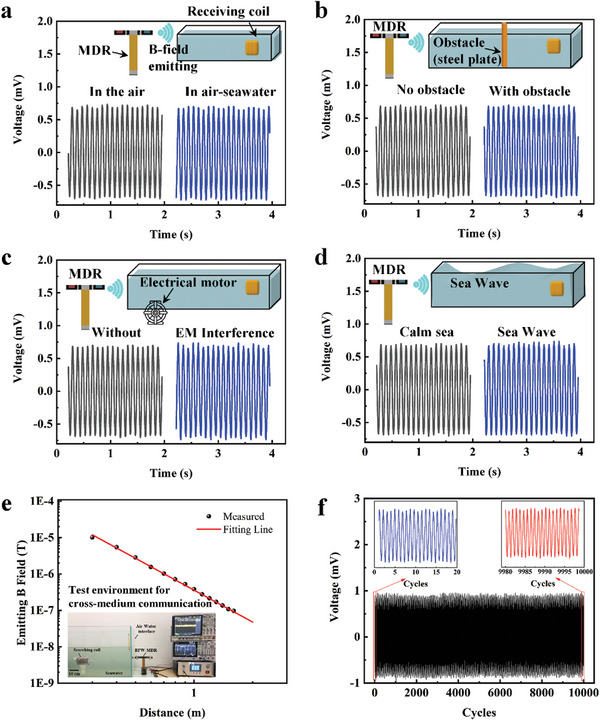
Demonstrates the immunity and stability of cross‐medium communication: a) signal voltage received both in air and water, b) A 5‐mm‐thick steel plate inserted in the electromagnetic wave's propagation path acts as a barrier in seawater, c) a working electromagnetic motor placed around the propagation path to simulate interference, d) harsh environment simulation with ocean waves, e) distribution of magnetic field strength for cross‐medium seawater transmissions, with an inset showing the test environment for cross‐medium communication, and f) signal strength measured at a distance of 1 meter from the resonator over 10 000 consecutive cycles.

In order to fairly evaluate the cross‐medium communication capabilities of the BFW‐MDR, we simulated an actual communication scenario by constructing a tank nearly two meters in length. The tank was filled with seawater, which has an electrical conductivity of ≈4 S m^−1^. The resonator was positioned immediately adjacent to the air‐sea interface. Additionally, a coil aligned with the direction of the electromagnetic wave emission (y‐axis) was submerged at various distances from the air‐sea interface. Details of the actual test setup are provided in the **Figure** **4**e. This placement of the resonator next to the air‐water interface minimizes air interference, preserving the integrity of the underwater magnetic signal strength.

Figure 4 illustrates the time‐domain signals generated by the resonator in air and the signals captured by the receiving coil in seawater at a distance of 1 meter. This includes: (i) Air‐seawater cross‐medium communication (Figure 4a), (ii) The impact of a 5 mm‐thick steel plate inserted into the propagation path (Figure 4b), (iii) The effects of operating a motor near seawater, which simulates electromagnetic interference (EMI) and vibration interference (Figure 4c), and (iv) Simulation of wave interference (Figure 4d).

In these scenarios, the received voltage signal shows minimal attenuation, highlighting the strong robustness of the ELF magnetic signal in harsh environments. Figure 4e observes that the decay pattern of the magnetic field strength when varying the underwater distance is almost identical to that in air, demonstrating the ELF flapping resonator's effectiveness in facilitating cross‐medium transmission. Additionally, Figure 4f demonstrates the stable performance of the mechanical antenna during operation, with the voltage collected by the search coil remaining extremely stable over 10 000 cycles. These observations collectively affirm that the BFW‐MDRS is both stable and possesses robust cross‐medium immunity.

### 2.5 Cross‐Medium Communication Demonstration

**Figure 5:**
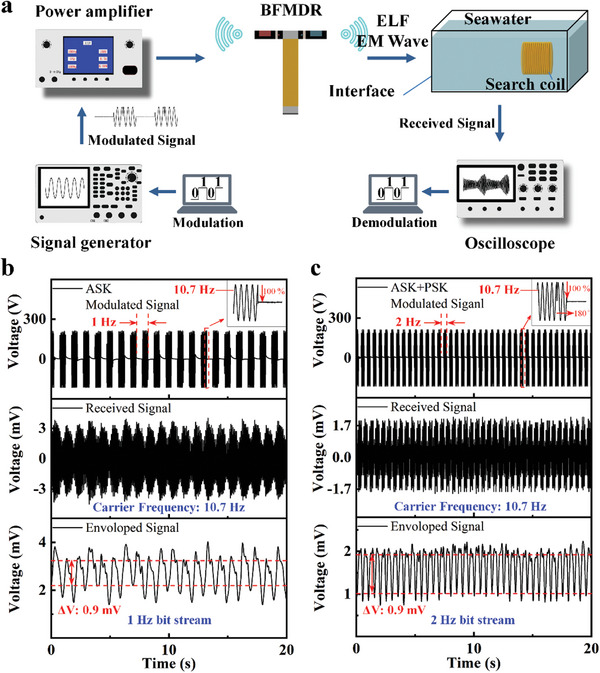
Demonstration of cross‐medium communication, showing the increase in communication rate. a) A schematic diagram of a cross‐medium communication system with BFW‐MDR mechanical antenna is shown. b) The ASK modulation communication mode with a modulation period of 1 Hz, and an envelope voltage ΔV of 0.9 mV, with the smaller ΔV, the higher the BER. The figure further illustrates the excitation voltage waveform, the received voltage waveform, and the demodulation signal, and the inset figure gives the details of the ASK modulation voltage variation; c) The ASK+PSK modulation communication mode, and the modulation period is raised to 2 Hz by using the same standard of ΔV value as that of the ASK envelope voltage. The inset gives details of the ASK+PSK modulation voltage variation, inverting the voltage at the end of the high level.

To assess the cross‐medium communication capability of the BFW‐MDR, an experiment was conducted wherein the receiving coil was positioned within a container filled with seawater. The coil was submerged at a location 50 cm away from the resonator in the air. **Figure** **5**a illustrates the schematic of the mechanical antenna communication system. Amplitude shift keying (ASK) and ASK combined with phase shift keying (PSK) baseband digital modulation schemes were employed to modulate the carrier signal, respectively. The modulated signal, generated using a signal generator (SG) and amplified through a power amplifier (PA). A modulating signal with a carrier frequency of 10.7 Hz (corresponding to the second‐order bending resonance frequency) was then applied to the mechanical antenna to facilitate programmable modulation of the radiated magnetic field signal.

For signal reception simulating cross‐medium communication, a search coil was placed in a seawater‐filled container, and the received signal was captured using either a lock‐in amplifier (LA) or a digital oscilloscope. It is noteworthy that the flapping resonator emits ELF radiation in air, highlighting the advantages of low‐frequency mechanical antennas in cross‐medium communication scenarios. Subsequently, we validated the ELF communication capability of the BFW‐MDR. The input signal undergoes ASK modulation, as illustrated in Figure 5b. The modulation period of the input voltage signal is set at 1 Hz. The signal captured by the coil reflects the waveform of the input signal. Next, the received signal undergoes demodulation to extract information from the modulated signal, effectively realizing a cross‐medium communication. It is noteworthy that with an increase in the modulation rate, the peak‐to‐peak value of the envelope signal, denoted as ΔV, diminishes. A smaller ΔV corresponds to a higher Bit Error Rate (BER).

The lower communication rate of flapping magnetic‐dipole resonator is due to the fact that the cantilever beam structure induces a substantial relaxation time (τ), impeding the signal modulation rate and resulting in an elevated BER. The relaxation time signifies the energy dissipation during the oscillations of the resonator when the excitation signal undergoes ON‐OFF transitions. Specifically, the measurements show that the relaxation time of the bionic flapping resonator is ≈4.8 s, representing the duration for an exponential decay from the peak value.^[^ ^16^ ^]^ The prolonged relaxation time adversely impacts the communication rate. To mitigate this challenge, a hybrid modulation approach combining ASK and PSK is employed. This method controls the 180‐degree phase shift by manipulating the baseband signal, which facilitates fast vibration suppression and thus effectively suppresses relaxation time. Here, we adopt the ΔV standard, identical to ASK, to elevate the communication rate to 2 Hz. This suppression contributes to an enhanced communication rate while concurrently mitigating the bit error rate associated with high‐speed transmission.

**Table** **1** provides a summary of performance parameters for existing mechanical antennas. The presented approach not only demonstrates the highest radiated magnetic field strength but also features a compact size, enhancing its suitability for subsequent antenna array configurations. The BFW‐MDRs structure, characterized by a high‐quality factor, enables the generation of a robust radiated magnetic field in the ELF range while maintaining ultra‐low power consumption. The power consumption of BFW‐MDR is less than one‐tenth that of an electromagnetic motor rotating magnet. Moreover, it exhibits minimal signal attenuation when transmitting through various medium, ensuring long‐term stability and offering an innovative solution for cross‐medium communication challenges. In our forthcoming research, we plan to fully optimize the piezoelectric phase characteristics and volume ratio to enhance EMM coupling and achieve stronger radiation performance with multiple single‐component arrays.

**Table 1:** Comparison of characteristics of different acoustic/magnetic resonators and mechanical antennas.

Method	V_a_ [cm^3^]	Freq [Hz]	Magnetic field intensity	P_in_ [W]	Bit rate [bps]	Quality factor [Q]	Ref.
EM motor	≈400	30	1.7 µT at 2.5 m	43.9		28	[22]
	58.5	22	600 nT at 1 m	0.36	8	8.23	[21]
	808.9	100	800 fT at 100 m	100	0.2	–	[3]
	18.8	440	0.1 uT at 1 m	0.35	214	14.6	[15]
	135	715	0.7 fT at 1 km	2	18	28.5	[33]
Linear resonator	2.26	10	6 nT at 1 m	0.027	–	–	[14]
	0.45	6300	1.12 nT at 0.5m	0.01	–	0.6	[26]
	1.8	18 100	3.8 nT at 0.5 m	0.11	1000	0.45	[27]
	4.5	21 600	108 fT at 100 m	–	300	–	[25]
	69	22 230	6 pT at 5.5 m	0.147	2000	0.05	[2]
	0.33	23 950	20 nT at 1 m	0.4	100	–	[23]
	50.3	33 230	40 fT at 6 m	1.2	100	–	[16]
	18.9	35 500	–	2800	>400	–	[17]
Rotating resonator	9.52	10	1.15 µT at 1 m	0.62	0.2	49	[19]
Swinging resonator	7	10.3	392 nT at 1 m	0.0033	–	1505^a)^	[28]
	19.92	6.86	514 nT at 1 m	0.0069	1	288	This work
	17.92	10.7	430 nT at 1 m	–	2	–	
Coil antenna	19	6.86	514 nT at 1 m	33		0.045	

a) In our previous report (Table 1 in ref. [28]), a factor of 2π was omitted in calculating Q‐factor of acoustic/mechanical resonators and rotating magnet. However, real Q‐factors should include 2π. For example, the Q‐factor for a swinging resonator in ref. [28] was only 240; while, real value should be as high as 1505 after including 2π.

## 3 Discussion

In conclusion, we introduce a novel solution for efficient transmission of ELF EM waves through the development of piezo‐actuated bionic flapping wing magnetic‐dipole resonator (BFW‐MDR). This bionic flapping‐wing design, incorporating a rigid‐flexible hybrid step‐stiffness wing reinforced and supported by magnetic force, emulates the flexibility observed in bird wings. This design not only mitigates the bending stiffness of the wings but also provides substantial rotational freedom, leading a tenfold amplification of the flapping oscillation of magnetic‐dipoles. The synchronized vibration of the two wings, each equipped with permanent magnets and oscillating in opposite phases, further facilitates the constructive superposition of the emitted magnetic fields, significantly enhancing the transmission of ELF EM waves.

Thus, the BFW‐MDRs exhibit an enhanced quality factor (Q) of ≈288 for transmitting ELF magnetic fields. Moreover, the resonator demonstrates an impressive emission of a magnetic flux density of 514 fT at a distance of 100 m, surpassing reported values in acoustic resonant mechanical antennas and showcasing a three‐order‐of‐magnitude improvement in ELF EM wave emitting efficiency compared to conventional loop coil antennas. To further enhance the communication capabilities of the BFW‐MDRs, we proposed an ASK+PSK modulation method, which not only suppresses the relaxation time but also doubles the communication rate to 2 bps, highlighting the versatility of our resonators for information transmission.

The robustness of the BFW‐MDRs is demonstrated through cross‐medium communication tests conducted between air and seawater under various external interferences, suggesting the BFW‐MDRs as promising, portable, highly efficient, and low‐power consumption antennas for applications in underwater and underground communications, showcasing their potential in diverse and challenging environments.

## 4 Experimental Section

The BFW‐MDRs consist of a piezoelectric phase, an aluminum alloy substrate, a pair of carbon fiber wings, tip mass, and two pairs of permanent magnets. The piezoelectric phase refers to a macro fiber composites (MFC) comprising PZT‐5H piezoelectric ceramic and epoxy resin, exhibiting dimensions of 100 × 20 × 0.3 mm. This composite was equipped with interdigital electrodes on both its upper and lower surfaces, enabling polarization in the longitudinal direction under a polarization voltage of 2 kV mm^−1^. The driving electric field was applied in the same length direction, enabling the piezoelectric phase to operate with a high d_33_ piezoelectric coefficient. The flexibility of the MFC enhanced the stability and lifespan of the resonator under high vibrational displacements. The aluminum substrate, measuring 126 × 22 × 0.5 mm, was bonded to the MFC using high‐strength epoxy resin to serve as a support beam for the resonator. One end of the beam served as the clamping end, while the other end served actuating end. One pair of flexible wings composed of carbon fibers equipped with NdFeB permanent magnets were symmetrically connected to the actuating end via two flexible polyimide film hinges, which were reinforced by magnetic repulsive force. This rigid‐flexible hybrid joint significantly enhanced the wing's vibration amplitude. NdFeB permanent magnets with a size of 7.2 cm^3^ in the two wings were magnetized in opposite directions, and permanent magnets at the flexible joints were mounted in the same direction as the magnetization of their respective sides on the two wings, and the joints of the crossbeam, respectively. The magnets at the connection generate repulsive forces between the two wings, thereby increasing the spreading ratio of the two wings, and the presence of synergistic effects due to the larger size of the permanent magnets in the wings results in an increase in the radiation intensity of the mechanical antenna. And a non‐magnetic tip mass with a size of 2 cm^3^ was attached on the cantilever beam to adjust the resonance frequency and B‐field radiation of the BFW‐MDR. The detailed fabrications are provided in the Section S9 (Supporting Information).

The experimental setup involved using a signal generator equipped with a power amplifier to apply an excitation voltage to the piezoelectric MFC. The near‐field magnetic field strength was subsequently detected using a circular search coil, and magnetic signals were acquired through either an oscilloscope or a lock‐in amplifier. Additionally, the vibration displacement of the BFW‐MDRs was continuously monitored by a high‐precision laser displacement sensor. This displacement data was then converted into a rotation angle by substituting the wing dimensions of the BFW‐MDRs.

## Conflict of Interest

The authors declare no conflict of interest.

## Author Contributions

Z.C. conceptualized the idea for the study; designed the methodology; performed investigation; visualized the idea for the study; performed validation; and wrote the original draft. L.M., Z.Q., B.W., Q.W., and W.C. performed formal analysis. All authors contributed to writing, reviewing, and editing the manuscript. S.D., J.Z., P.C., and J.C. performed project administration; acquired resources; and performed funding acquisition.

## Supporting information

Supporting Information

## Data Availability

The data that support the findings of this study are available from the corresponding author upon reasonable request.
