# Supplementary material for: A Bionic Flapping Magnetic‐Dipole Resonator for ELF Cross‐Medium Communication
Source: Adv Sci (Weinh). 2024 Jun 14;11(30):2403746. doi: 10.1002/advs.202403746 (PMC11321680; doi:10.1002/advs.202403746)
Supplement: Supplementary file 1 — Supporting Information [file ADVS-11-2403746-s001.docx]

## Supporting Information

# A Bionic Flapping Magnetic-Dipole Resonator for ELF Cross-Medium Communication

*Zhi Cheng*^a,b^, *Jing Zhou*^a,*^, *Bin Wang*^b^, *Qiong Wu*^b^, *Liang Ma*^a^, *Zhi Qin*^a^, *Jie Shen*^a^, *Wen Chen*^a^, *Wei Peng*^b^*, Jianglei Chang* ^c,*^, *Penghong Ci* ^d,*^*, and Shuxiang Dong*^b,*^

**S1. Analysis of the mechanical quality factor (Q_m_) of piezoelectric resonators and the quality factor (Q) of magnetic dipole antennas.**

The evaluation of resonant coupling in a resonant system is closely associated with the high mechanical quality factor (Q_m_) of the composite material, typically determined by the 3 dB bandwidth of the resonant peaks. The mechanical quality factor is calculated using the formula:

$Q_{m}=f_{r}/\Delta f$ （S1）

where f_r_ is the resonant frequency and Δf is the 3 dB bandwidth of the magnetoelectric (ME) composite. Based on the frequency domain data presented in Figure 2d, the mechanical quality factor Q_m_ of the flapping-wing resonator is determined to be 19.6, which is notably high for a low-frequency resonant system.

In the context of this study, where the flapping-wing resonator functions as a magnetic dipole mechanical antenna, we utilize the static magnetic field theory proposed for near-field conditions to evaluate the communication efficiency in magnetic near-field communication. We use the quality factor Q of the resonator antenna as a metric for assessing the efficiency of near-field communication. As we explained in (Adv. Mater. 2024, 36, 2309159) article, the definition of Q is based on the alternating magnetic field energy at steady state:

$Q=\frac{\omega max\left\{ W_{m},W_{e} \right\}}{P_{\mathrm{Loss}}}$ （S2）

where ω is the angular frequency, W_m_ is the magnetic field energy (the magnetic field energy W_m_ far surpasses the electric field energy W_e_ in the near field), and $P_{Loss}=\omega C\left( Et_{p} \right)^{2}PF$ includes both ohmic and mechanical losses of the resonator. Since the oscillation of a pair of permanent magnets can be equated to an oscillating magnetic dipole moment, the maximum stored energy of the alternating magnetic field in the airspace, W_m_ is

$W_{m}=\frac{1}{2}\text{∰}_{r_{a}}^{\infty}BHdV=\frac{\mu_{0}\Delta m^{2}}{12\pi r_{a}{}^{3}}$ （S3）

where r_a_ is the effective antenna radius.

When the magnetic dipole mechanical antenna operates in acoustic resonance, the input power Pin of the resonator under an applied electric field E is significant for the overall system's functionality and efficiency.

Therefore, combining equations (S1) and (S2), we get

$Q=\frac{\left( \alpha B_{r}V_{m} \right)^{2}}{9V_{a}\mu_{0}Ct_{p}^{2}PF}$ （S4）

where C represents the capacitance of the piezoelectric phase, PF denotes the power factor, t_p_ is the electrode spacing, B_r_ is the remanent magnetization, V_m_ is the volume of the permanent magnet, and V_a_ is the volume of the antenna. Additionally, α is defined as the piezoelectric actuation coefficient, with (β = αE), indicating that increasing the driving electric field E of the resonator enhances the rotation angle β of the permanent magnet.

Based on the principle of enhancing radiation efficiency by increasing β (the variation of magnetic dipole moment) and reducing PF (power factor, indicative of lower losses and a higher mechanical quality factor, Q_m_), we conduct theoretical and experimental validations. These studies analyze how the resonance modes of the piezoelectric beam influence the vibration of wings and the radiation of electromagnetic waves. This analysis is crucial for optimizing the design and functionality of the resonator in practical applications.

**S2. FEA simulation model**

As shown in Fig. S1a, the rotating setup and magneto-physical field are established to study the changes of magnetic vector position and magnetic flux density in space when the permanent magnet rotates. A permanent magnet with a length, width and height of 20×10×5 mm is located at the origin of coordinates. The Fig. S1 shows multiple sections and streamlines of the air domain magnetic flux density, and the color legend represents the magnetic flux intensity.

In order to simulate the motion mode of the permanent magnet in the flapping wing resonator, the permanent magnet was magnetized toward the z axis and rotated around the x-axis. By collecting the magnetic flux density at the coordinate point (0, 1 m, 0) and changing the flapping rotational angle of the permanent magnet, the magnetic flux density relationship corresponding to different rotation angles was obtained, as shown in Fig. S1b. It is verified that higher space magnetic field intensity can be obtained by increasing the flapping rotational angle of permanent magnet and increasing the change of magnetization.

**
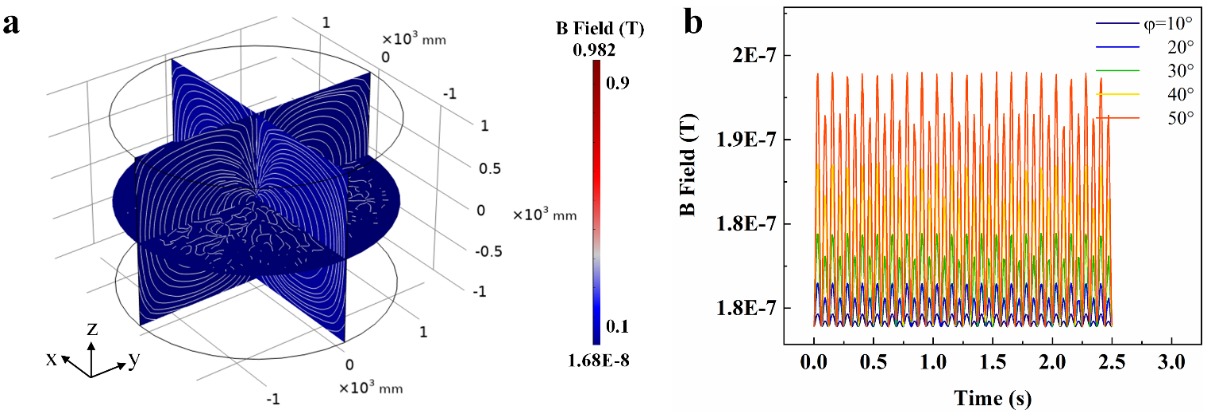
**

Fig. S1 **a** Physical field model of rotating permanent magnet; b The relationship between the rotation Angle of permanent magnet and the magnetic field strength.

**S3. Performance comparison of flapping wing resonators with different vibration modes.**


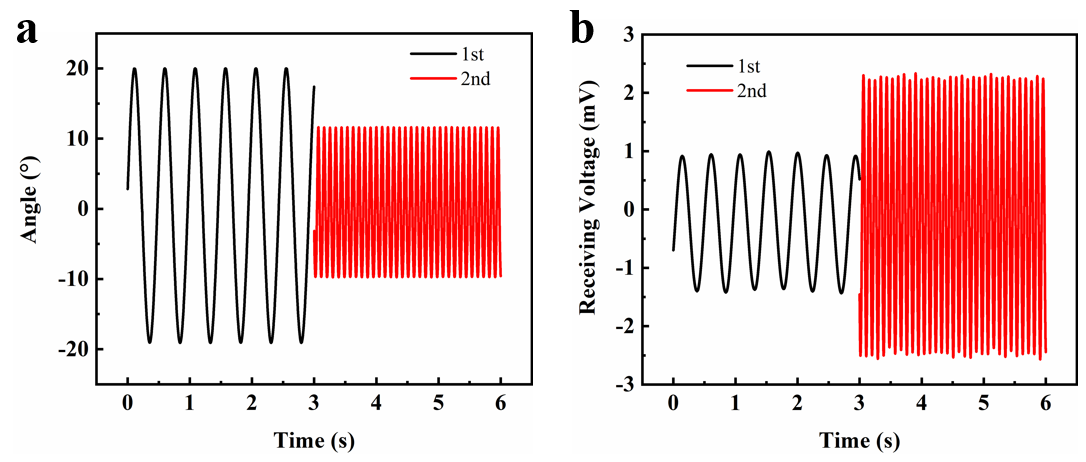


Fig. S2 **a** The comparison between the overall flapping rotational angles for the first-order bending mode and the second-order flapping mode; **b** The output voltage waveform comparison for the two vibration modes generated by the search coil.

**S4. The performance of a flapping wing resonator with same magnetization direction.**

To verify this theory, we established a contrast structure using the same basic structure as that of the main text's flapping-wing resonator antenna. However, we modified the permanent magnets at the ends of the beam to ensure that both wings are magnetized in the same direction, and placed the same direction permanent magnets at the end of the beam for confinement. Figures S1a and b depict the schematic and the physical objects, respectively. As shown in Figure S1c, the opposite direction magnetized resonator antenna achieves the maximum radiated magnetic field strength at the second-order bending resonance. Since the magnetization directions are the same, the magnetic dipole moments along the r-direction (y-axis) cancel out. Figure S1d inset shows that the maximum radiation intensity is directed along the x-axis. Here, the magnetic field strength is 314 nT@1 m, attributed to the magnetic field component in the $\boldsymbol{\theta}$ direction, $\boldsymbol{B}_{\theta}=\frac{\mu_{o}m_{o}}{4\pi r^{3}}(\sin\theta\boldsymbol{\theta})$. It can be found that the magnetic field strength $\boldsymbol{B}_{r}=\frac{\mu_{o}m_{o}}{2\pi r^{3}}(\cos\theta\boldsymbol{r})$ along the $\boldsymbol{r}$-direction (y-axis) of the structure used in the main text is about 514 nT@1 m, nearly twice as much as that in the flapping resonator with opposite magnetization direction.

After constructing the opposite magnetization direction flapping resonator as a comparison, we rigorously validated the exceptional performance of the bionic flapping resonator introduced in this study, employing both theoretical analysis and experimental testing.


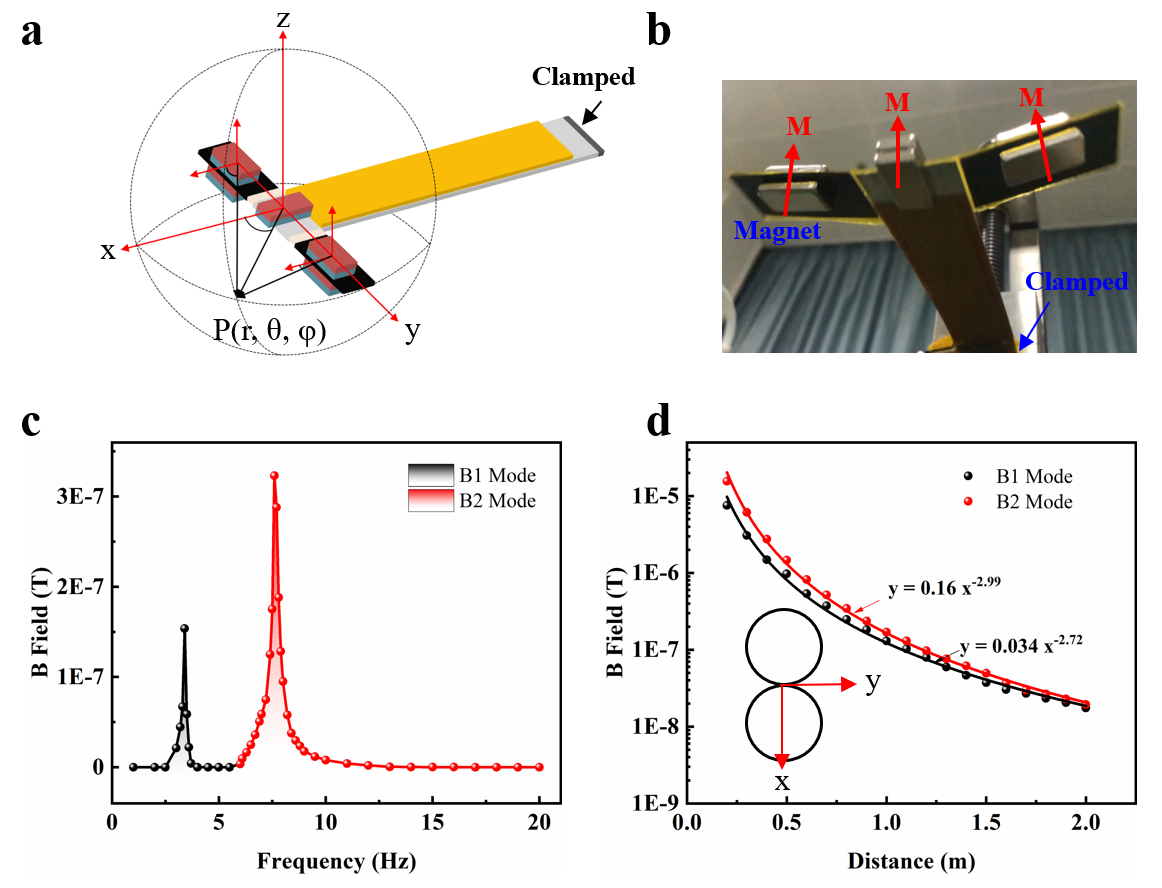


Fig. S3 illustrates the performance of a flapping wing resonator with same magnetization direction. **a** presents a schematic of a bionic flapping wing resonator featuring permanent magnets on each wing magnetized in the same direction, with additional permanent magnets positioned at the end of the beam, serving as 'magnetic constraints' on the wing. **b** showcases photographs of the resonator. **c** and **d** depict the frequency-domain and distance characteristics of the emitted magnetic field generated by the resonator.

**S5. The received signal V_r_ from the receiving coil after the incorporation of the tip mass.**


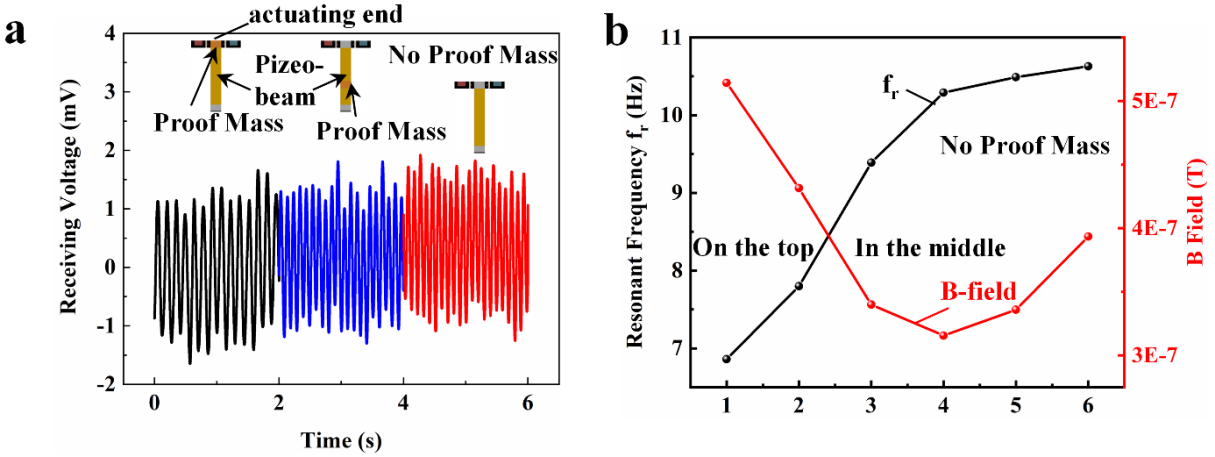


Fig. S4 **a** The change of the additional tip mass’s position enables a stepless adjustment of resonance frequency; **b** illustrates the correlation between the position of the proof mass, the natural frequency, and the radiation magnetic field intensity

**S6. Nonlinear analysis between radiated magnetic field and voltage.**

Figure 3c illustrates the nonlinear relationship between the radiated magnetic field and the voltage across the resonator. This nonlinearity arises from the innovative introduction of magnetic confinement and hinge connections. In the resonator's flexible structure, the oscillation angle of the permanent magnet lags behind the voltage changes due to inertia. As the piezoelectric amplitude increases, this lag causes a nonlinear effect between the drive voltage and the radiated magnetic field.

Further analysis of the system's nonlinearity from the perspective of magnetic constraints reveals interactions involving the permanent magnets (m_r_) located on two wings. These magnets are repelled by the gradient ***B(r)*** of the dipole field, which is generated by another magnetically confined permanent magnet (m_f_) positioned at a distance (d). The magnetic moments of m_r_ and m_f_ are represented by m = VM, and the magnetic field gradient ***B(r)*** is given by B(r) = m/2πr^3^. The magnitude of the magnetic force, which influences the wing motion, is a crucial factor in understanding the nonlinear dynamics of the system.

The magnetic force magnitude

$F_{m}(d)=\left| \nabla\left( \boldsymbol{m}\cdot\boldsymbol{B}(\boldsymbol{r}) \right) \right|=\frac{\boldsymbol{V}_{r}\boldsymbol{V}_{f}}{2\pi d^{4}}M^{2}cos\beta$ (S5)

where the volume is V, the residual magnetization strength M=1.4 T/μ_0_ and β is the angle at which the permanent magnet deviates from the equilibrium direction. Given that the magnetic constraint force provided by m_f_ is quartic with respect to distance, the resonator behaves as a nonlinear system. By rationally designing the relative distances between m_f_ and m_r_, a strong magnetic constraint force is generated, maintaining the stable periodic oscillations of the permanent magnets m_r_ on both wings.

**S7. The intensity of the transmitting magnetic field of the coil antenna.**

For a fair comparison of radiation efficiency between the flapping-wing resonator and a conventional loop antenna, we prepared a loop coil antenna with a comparable volume of 19 cm³. The radiation characteristics of the loop antenna were modeled similarly to a magnetic dipole, with the magnetic dipole moment $\Delta m=I_{0}\mathrm{SN}\mu_{0}$. Our analysis shows that the magnetic dipole moment for the flapping-wing resonator can be expressed as $\Delta m=B_{r}V_{m}sin\beta/\mu_{0}$. For an equitable comparison, when both the loop antenna and the flapping-wing resonator are of the same size and have the same magnetic dipole moment, they produce comparable effects.

Using equation (2), we calculated the magnetic dipole moment Δm = 2.57 Am². To generate this magnetic dipole moment, a loop antenna would require an input power of 33 W to achieve a magnetic field strength of 514 nT@1 m. Experimental results, as shown in Fig. S3, indicate that at a very low frequency of 6.86 Hz, the loop antenna requires an input power of 33 W to match the 514 nT field strength emitted by the BFW-MDR at 1 meter. In stark contrast, the flapping-wing resonator operates with an input power of only 0.0069 W. This demonstrates that the BFW-MDR antenna is approximately 4782 times more efficient in radiation than the conventional loop antenna.





Fig. S5 displays the emitting magnetic field strength (B) against input power for a coil antenna of comparable size (19 cm³).

**S8. Cross-medium communication experiments in real application scenarios.**

Furthermore, we have conducted communication experiments in actual pool water, as reviewer suggested, obtaining results that are largely consistent with those observed in laboratory scenarios.


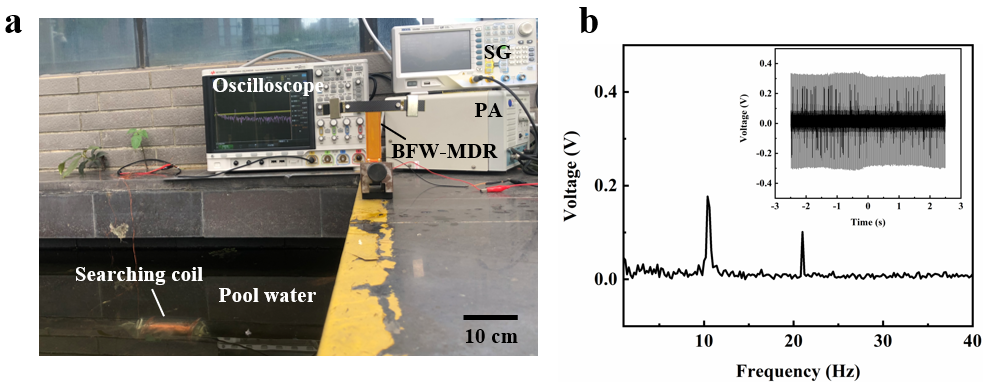


Figure S6 **a** Communication test under pool water; **b** Time and frequency domain signals received by the searching coil.

**S9. Preparation processes of Bionic Flapping-Wing Magnetic-Dipole Resonator (BFW-MDR) device**

Fabrication of the Macro Fiber Composites (MFC): The fabrication of Macro Fiber Composites (MFC) involved multiple stages, beginning with the preparation of Pb(Zr_0.53_Ti_0.47_)O_3_ (PZT-5H) piezoelectric ceramic blocks using traditional solid-state reaction method. For the construction of axially driven MFCs, the blocks underwent a series of processes including grooving, potting with epoxy resin and slicing into piezoelectric composite plates. A vacuum hot-pressing method was employed to encapsulate these plates with interdigital (ID) electrodes with an electrode spacing of 0.5 mm. Finally, the MFC was polarized under an electric field of 2 kV/mm. The driving electric field was oriented in the lengthwise direction, enabling the piezoelectric phase to operate in the d_33_ mode. This structural configuration and the fabrication method enhance the flexibility of the MFC, significantly improving the stability and operational lifespan of the resonator, particularly under conditions of high vibration displacements.

Fabrication of the BFW-MDR device: The fabrication of the BFW-MDR device involves constructing a flapping wing resonator that includes several components: an MFC, an aluminum alloy beam, a carbon fiber wing, a tip mass block, two pairs of magnetically confined permanent magnets (m_f_) and permanent magnets (m_r_) attached to the wing. Detailed structural dimensions and material properties are provided in the Table S1, S2.

The assembly process started with the MFC being bonded to the aluminum substrate using high-strength epoxy resin. The beam was designed with one end as the clamping end and the opposite end as the actuation end. The carbon fiber wing was then attached to this actuated end, ensuring symmetrical alignment in the width direction. A flexible hinge structure was created at the connection of the carbon fiber wing and the beam using a flexible polyimide film. At the actuated end of the beam, NdFeB permanent magnets (designated as m_r_) were attached to the wing and were magnetized in opposite directions. Additionally, a pair of permanent magnets (m_f_) was positioned 12 mm away from these wing magnets. The mf magnets, located at the connection, generate a repulsive force between the two wings. This force can be carefully adjusted based on the distance and volume of the magnets to optimize performance. This magnetic configuration not only increased the spreading ratio of the wings but also enhanced the stability of the resonator. Additionally, a non-magnetic proof mass measuring 2 cm³ was attached to the cantilever beam. This modification served to adjust the resonant frequency and enhance the B-field radiation of the BFW-MDR. The combination of rigid and flexible connections significantly boosts the vibration amplitude of the wing. This increased amplitude, coupled with the anisotropic magnetization of the permanent magnets in the wing, results in a notable enhancement of the radiation intensity of the mechanical antenna due to the magnetic field superposition effect.

Table S1. Information on the material properties of the flapping-wing resonators.

| Materials/Parameters | Young modulus  C_33_ (GPa) | Density  ρ (g/cm^3^) | Piezoelectric property  d_33_ (pm/V) | Remanent magnetization  Br (T) |
| --- | --- | --- | --- | --- |
| MFC | 35 | 5.7 | 437 | -- |
| Magnet | 170 | 4.5 | -- | 1.4 |
| Aluminum beam | 70 | 2.6 | -- | -- |
| Polyimide film | 1.8 | 1.23 | -- | -- |
| Epoxy resin | 3.1 | 1.2 | -- | -- |
| Carbon fiber | 230 | 1.5 | -- | -- |
| Proof mass (Cu) | 110 | 8.96 | -- | -- |

Table S2. Structural and dimensional information for flapping resonators

| Materials | Rigid connections resonator  Size L*W*T (mm) | Magnetic constraints resonator  Size L*W*T (mm) |
| --- | --- | --- |
| MFC | 100*20*0.3 | 100*20*0.3 |
| Magnet (mr) | 40*20*3 | 40*20*3 |
| Magnet (mf) | -- | 2*5 (R*T) |
| Aluminum beam | 126*22*0.5 | 126*22*0.5 |
| Carbon fiber 1 | -- | 40*20*0.5 |
| Carbon fiber 2 | 190*20*0.5 | 110*20*0.5 |
| Proof mass (Cu) | -- | 20*20*5 |
